# *aflN* Is Involved in the Biosynthesis of Aflatoxin and Conidiation in *Aspergillus flavus*

**DOI:** 10.3390/toxins13110831

**Published:** 2021-11-22

**Authors:** Kunzhi Jia, Lijuan Yan, Yipu Jia, Shuting Xu, Zhaoqi Yan, Shihua Wang

**Affiliations:** Key Laboratory of Pathogenic Fungi and Mycotoxins of Fujian Province, Key Laboratory of Biopesticide and Chemical Biology of Education Ministry, School of Life Sciences, Fujian Agriculture and Forestry University, Fuzhou 350002, China; kjia@fafu.edu.cn (K.J.); lyan@fafu.edu.cn (L.Y.); yipujia@fafu.edu.cn (Y.J.); shutingxu@fafu.edu.cn (S.X.); zhaoqiyan@m.fafu.edu.cn (Z.Y.)

**Keywords:** *Aspergillus flavus*, AflN, aflatoxin, conidiation

## Abstract

*Aspergillus flavus* poses a threat to society economy and public health due to aflatoxin production. *aflN* is a gene located in the aflatoxin gene cluster, but the function of AflN is undefined in *Aspergillus flavus*. In this study, *aflN* is knocked out and overexpressed to study the function of AflN. The results indicated that the loss of AflN leads to the defect of aflatoxin biosynthesis. AflN is also found to play a role in conidiation but not hyphal growth and sclerotia development. Moreover, AlfN is related to the response to environmental oxidative stress and intracellular levels of reactive oxygen species. At last, AflN is involved in the pathogenicity of *Aspergillus flavus* to host. These results suggested that AflN played important roles in aflatoxin biosynthesis, conidiation and reactive oxygen species generation in *Aspergillus flavus*, which will be helpful for the understanding of *aflN* function, and will be beneficial to the prevention and control of *Aspergillus flavus* and aflatoxins contamination.

## 1. Introduction

*Aspergillus flavus* (*A. flavus*) is a notorious pathogenic fungus, which can produce aflatoxins (AFs) and contaminates many crop seeds, leading to the large economic losses [1]. It is worth noting that *A. flavus* is typically found in soil and distributed worldwide due to its strong survival capability [1,2]. Therefore, *A. flavus* poses a threat to society economy and public health. A lasting and deep study on *A. flavus* will help us better understanding and controlling of *A. flavus* and AFs. As the main focus of *A. flavus* study, biosynthesis pathway of AFs is constituted of more than 25 enzymatic reactions [3,4], and these enzyme genes are mainly clustered on chromosome 3 [5]. Many other genes outside the AF gene cluster also have effects on the biosynthesis of AF [6,7]. CreA, which is the master regulator of carbon catabolite repression, was found to regulate the AF biosynthesis and conidia development.

*aflN,* located at the aflatoxin (AF) gene cluster, is a member of cytochrome P450 family [8]. Previous reports suggested that *aflN* in *A. parasiticus* and *A. nidulans* is involved in the biosynthesis of aflatoxins (AFs), but the detailed functions are still undetermined [3,9]. Genetic disruption of *stcS*, a homolog of *aflN* in *A. nidulans*, led to the accumulation of versicolorin A (VA) and blocked the formation of sterigmatocystin (ST) [9,10], which is an important step for biosynthesis of aflatoxin. Although AflN is suggested to be involved in the biosynthesis of aflatoxin, the direct evidence of AflN involved in AF biosynthesis in *A. flavus* is absent, and the potential other function for AflN is still unclear. In this study, genetic *aflN* mutants were constructed with homology recombination, and the effects of *aflN* on the development and metabolism were then investigated for better understanding of AflN biofunctions in *A. flavus*.

## 2. Results

### 2.1. Identification and Analysis of AflN in A. flavus

**Figure 1:**
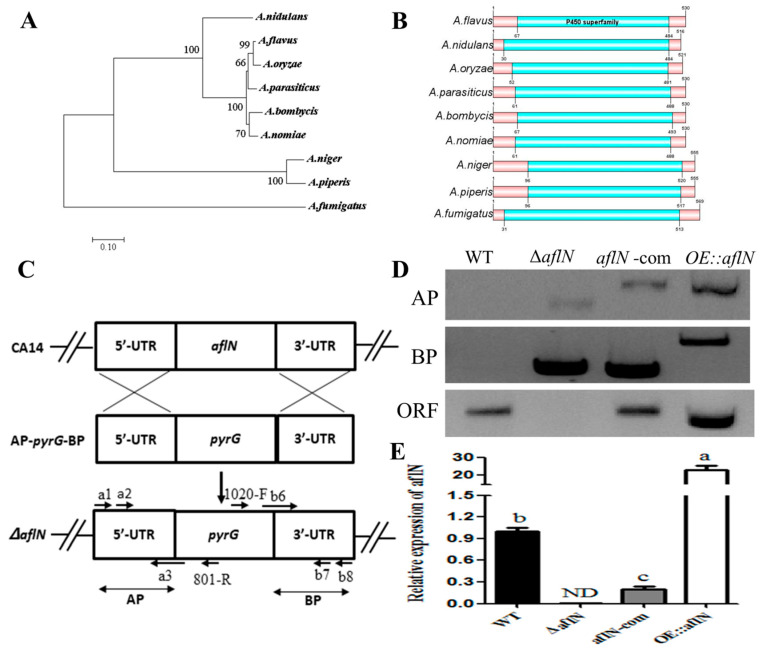
AflN identification and mutant strains construction. (**A**) Phylogenetic tree of AflN homologous proteins from various fungi. The phylogenetic tree was constructed using MEGA X with protein sequences. (**B**) Conserved domain analysis of AflN homologous proteins in different species. (**C**) A typical schematic describing the disruption strategy in this study. UTR represents untranslation region. AP and BP represent A homology arm part and B homology arm part respectively. (**D**) *aflN* mutant strains were verified with PCR. WT means wild type. AP and BP represent A homology arm part and B homology arm part respectively. ORF represents open reading frame of *aflN* gene. (**E**) Expression of *aflN* was examined by qRT-PCR in different *A. flavus* strains. ND means not detected. Different lowercase letters above the bars represent significant difference (*p* < 0.05).

*A. flavus* AflN protein was identified from the National Centre for Biotechnology Information (NCBI) database with the sequence ID: XP_002379939.1 (G4B84_005799). Protein sequences from 9 fungi were aligned using MEGA X, and then a phylogenetic tree was constructed. As shown in Figure 1A, *A. flavus* AflN protein shares a 99% similarity with *A. oryzae* AflN, and high similarity with homologues from other 8 fungi, showing that AflN probably plays a similar role in these species. *A. flavus* AlfN has a P450 superfamily domain, highly similar to P450 monooxygenases from other fungi (Figure 1B). To further study the function of AflN, *aflN* knockout (Δ*aflN*), complementary (*aflN-com*) and overexpression (*OE::aflN*) strains have been constructed using homology rearrangement strategy (Figure 1C). The strains’ genotyping were verified with PCR method (Figure 1D). Expression levels of *aflN* in various strains were confirmed using qRT-PCR (Figure 1E), showing that *aflN* mutant strains were successfully constructed for further function study.

### 2.2. AflN Plays a Role in the Aflatoxin Biosynthesis in Cytoplasm

**Figure 2:**
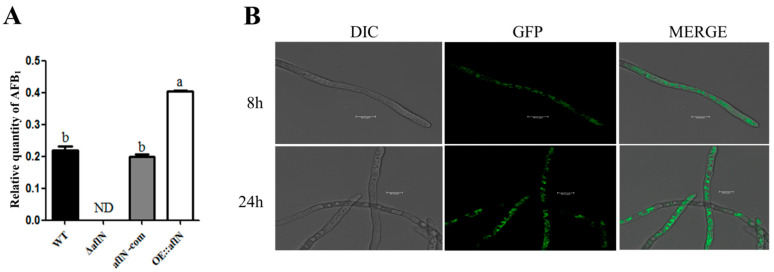
AflN was involved in biosynthesis of aflatoxins. (**A**)The relative quantity of aflatoxins AFB1 in *WT, ΔaflN, aflN-com* and *OE::aflN*. ND means not detected. Different lowercase letters represent significant difference (*p* < 0.05). (**B**) Location of AflN in *A. flavus* by examing *alfN-gfp* expression with fluorescent microscopy. DIC means differential interference contrast. GFP represents green fluorescent protein.

To study the effect of AflN on the biosynthesis of AFB1, aflatoxin levels have been assayed in various strains by TLC (thin layer chromatography) method. As shown in Figure 2A, compared with WT and *aflN-com*, AFB1 was barely detected in Δ*aflN* but significantly increased in *OE::aflN*, showing that AflN plays a positive role in the aflatoxin biosynthesis. As known, the location of protein is related with its bio-function. To examine the distribution of AflN, *aflN* fusing with *gfp* was inserted in chromosome using homology rearrange method. As shown in Figure 2B, AflN was distributed in the cytoplasm at 8 h post inoculation of conidia. To make clear whether AflN changed its location at hyphal growth stage, the location of AflN at 24 h post inoculation was also examined. As shown in Figure 2B, AflN was still distributed in the cytoplasm at 24 h. These results indicated that AflN played a critical role in the biosynthesis of AFB1 in cytoplasm.

### 2.3. AflN Is Involved in Conidiation but Not Hyphal Growth and Sclerotia Development

**Figure 3:**
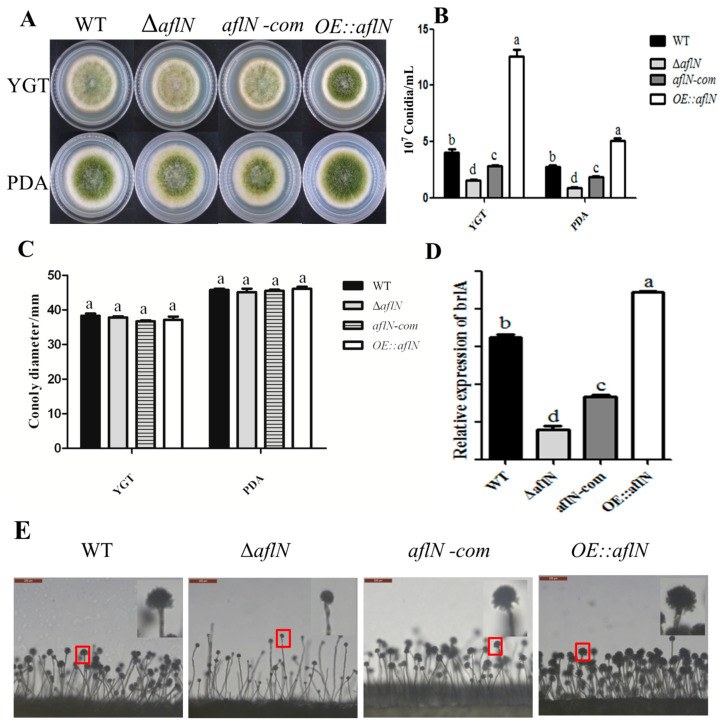
The role of AflN in conidiation and hyphal growth. (**A**) Colony morphology of the WT, Δ*aflN*, *aflN*-*com* and OE::*aflN* strains grown on YGT and PDA. (**B**) The comparison of conidia number in WT, Δ*aflN*, *aflN*-*com* and OE::*aflN*. (**C**) The comparison of hyphal growth in WT, Δ*aflN*, *aflN*-*com* and OE::*aflN*. (**D**) *BrlA* expression in WT, Δ*aflN*, *aflN*-*com* and OE::*aflN*. (**E**) Conidiophore morphology of WT, Δ*aflN*, *aflN*-*com* and OE:: *aflN* strains of *A. flavus* grown on PDA. Different lowercase letters represent significant difference (*p* < 0.05).

**Figure 4:**
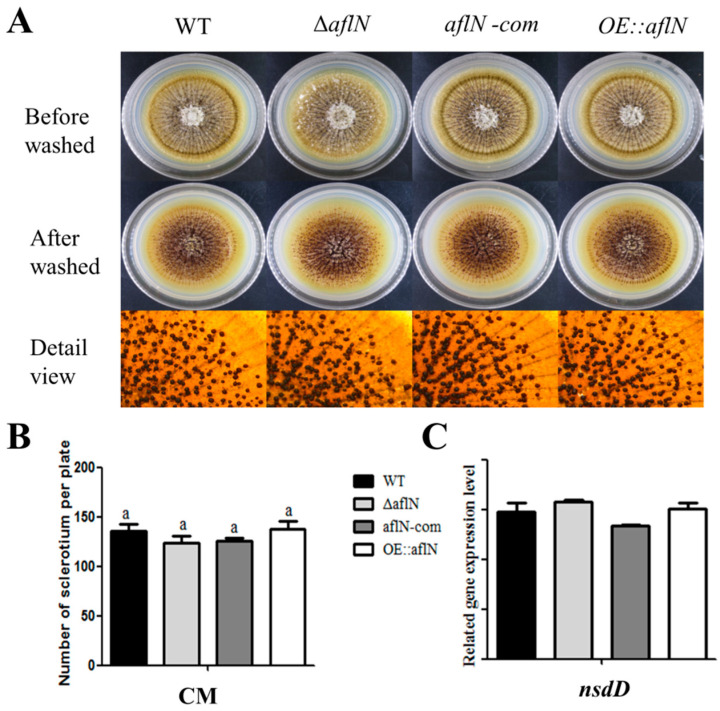
Sclerotia analysis of *A. flavus* among WT, Δ*aflN*, *aflN*-com and *OE::aflN* strains. (**A**) Phenotypes of WT, Δ*aflN*, *aflN*-com and OE::*aflN* strains were determined on CM (complete medium) medium. (**B**) The comparison of sclerotia number among WT, Δ*aflN*, *aflN*-com and OE::*aflN* strains. The lowercase letter “a” represents no significant difference (*p* > 0.05) among various strains. (**C**) Expression levels of *nsdD* involved in sclerotia production by qRT-PCR.

To study the role of AflN in conidia development, conidiation was studied in various strains. As shown in Figure 3A, the morphology exhibits difference among WT, Δ*aflN*, *aflN-com* and *OE::aflN*. At the same time, the conidia number was significantly decreased in Δ*aflN* but increased in *OE::aflN*, compared with WT and *aflN-com*. This phenomenon was observed both in YGT (yeast extract, glucose and trace elements) and PDA (potato dextrose agar) medium (Figure 3B), which indicated that AflN played an important role in the conidia development. As *brlA* gene plays a critical role in the conidiation [11], *brlA* expression has been examined in this study. As in Figure 3D, *brlA* expression was decreased in Δ*aflN* but increased in *OE::aflN*, compared with WT and *aflN-com*. At the same time, conidiophores in various strains have been observed. As shown in Figure 3E, less conidia and poorly developed conidiophores were observed in Δ*aflN*. These results suggested that the decreased conidiation in Δ*aflN* may be due to the decreased *brlA* expression and poor conidiophores. To investigate whether AflN plays a role in other developmental processes, the hyphal growth was also been examined. The colony diameters were measured, and the result in Figure 3C showed that there is no difference among Δ*aflN*, *aflN-com* and *OE::aflN*. Sclerotia development was also assayed among various strains to determine the role of AflN in this process. As shown in Figure 4A,B, equal levels of sclerotia were observed among Δ*aflN*, *aflN-com* and *OE::aflN* strains. The expression of sclerotia related gene, *nsdD*, exhibited a similar level among various strains (Figure 4C). Thus, AflN has no effect on sclerotia development. All of the above results indicated that AflN has been involved in conidiation but not hyphal growth and sclerotia development in *A. flavus*.

### 2.4. Absence of aflN Leads to Less Response to Oxygen Stress

**Figure 5:**
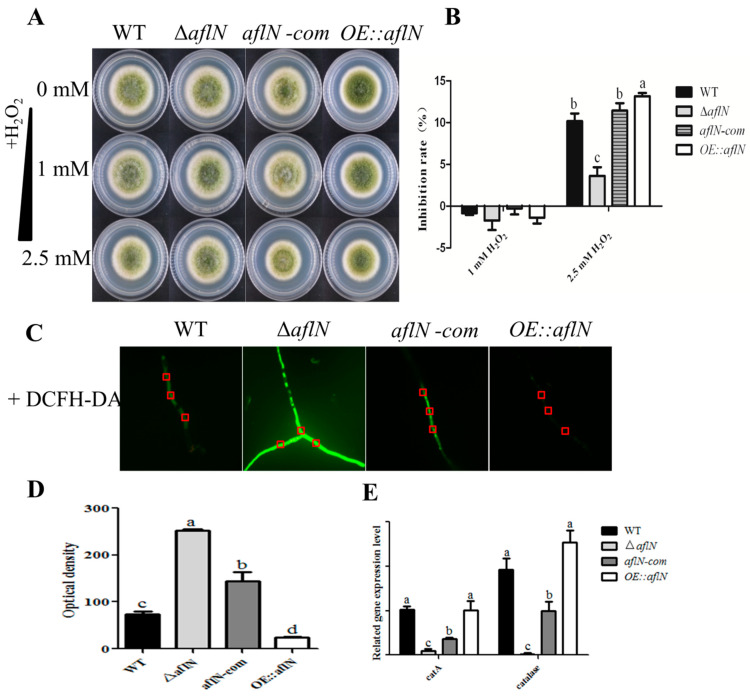
The role of *aflN* in oxidative stress. (**A**) Strains were treated with 1 mM H_2_O and 2.5 mM H_2_O_2_ stress. (**B**) The comparison of the growth inhibition rate among WT, Δ*aflN*, *aflN*-com and *OE::aflN* strains treated with H_2_O_2_. (**C**) ROS signal was indicated with DCFH-DA staining in various strains. (**D**) Quantitative analysis of ROS signals in various strains. (**E**) Expression levels of oxidation-related enzyme genes (*catalase like* and *catA*) in various strains. Different lowercase letters represent significant difference (*p* < 0.05).

As cytochrome P450 enzyme, AflN is closely related to the oxygen-reduction system [12]. To study the role of AflN in oxidative stress response, growth of *A. flavus* has been examined in various strains with H_2_O_2_. As shown in Figure 5A,B, Δ*aflN* strain showed less responsible to 2.5 mM H_2_O_2_ than WT and *aflN-com*; in contrast, *OE::aflN* showed more sensitive to H_2_O_2_, indicating that aflN is involved in the response to oxygen stress. Intracellular levels of reactive oxygen species (ROS) reflect the state of oxygen stress to a certain extent [13]. Therefore, ROS levels were further examined using DCFH-DA (6-carboxy-2,7-dichlorodihydrofluorescein diacetate) staining in this study [14]. As shown in Figure 5C,D, the ROS levels were significantly higher in Δ*aflN* than WT and *aflN-com*. In contrast, the ROS levels were lower in *OE::aflN* strain. To study the reason for high ROS in Δ*aflN*, the expression levels of ROS related genes have been examined. As shown in Figure 5E, compared with WT and *aflN-com*, the expression levels of ROS scavenging enzyme genes, *catalase-like* and *catA*, were significantly decreased in Δ*aflN* but increased in *OE::aflN*. This phenomenon indicated that the ROS scavenging enzymes are related to the increased levels of ROS in Δ*aflN*.

### 2.5. AflN Is Important for A. flavus Pathogenicity to Crops Seeds

**Figure 6:**
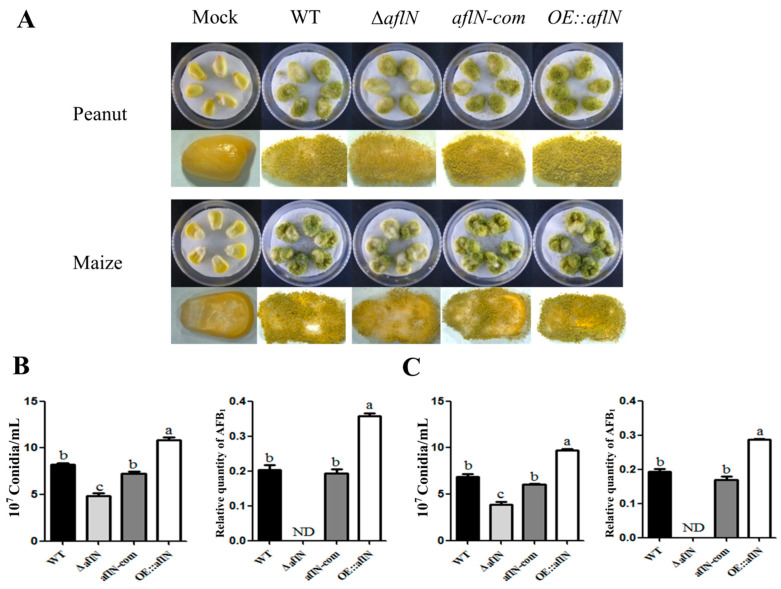
The effect of *aflN* gene on the pathogenicity of *A. flavus*. (**A**) Infection of peanuts and maize with various strains. Mock represents a control without any AF strain’s infection. (**B**) The number of conidia and the relative quantity of AFB1 in infected peanuts. (**C**) The number of conidia and the relative quantity AFB1 in infected maize. Different lowercase letters represent significant difference (*p* < 0.05).

In this study, the pathogenicity of *A. flavus* was assayed by the infection to peanuts and maize [15]. As shown in Figure 6A, the infections of peanuts and maize were observed at the 5th day post-inoculating, and the result indicated that the infection in Δ*aflN* was less than that in WT and *aflN-com*, but the infection in OE::*aflN* was more than that in WT and *aflN-com*. The aflatoxin levels have also been measured by TLC. As shown in Figure 6B,C, compared with WT and *aflN-com* strain, AFB1 in Δ*aflN* infecting peanuts and maize was barely detected, but AFB1 in *OE::aflN* infections significantly increased. At the same time, conidia numbers from the infected peanuts and maize were quantified. As shown in Figure 6B,C, conidia from Δ*aflN* infecting peanuts and maize were significantly less than that in WT and *aflN-com* strain, while conidia from peanuts and maize infected with *OE::aflN* were more than that in WT and *aflN-com* strain. These results indicated that AflN is important for *A. flavus* pathogenicity to crops seeds, and absence of AflN leads to the impairment of pathogenicity.

## 3. Discussion

Genetic disruption of *stcS* was found to block the conversion of VA to ST in *A. nidulans* [9]. Then, *StcS* was suggested to be a homology of *verA* (*aflN*) based on the high similarity of their protein sequences [3,10]. VerA (AflN) in *A. parasiticus* is proposed to be involved in the conversion of VA to ST, one of undefined steps before ST formation. In this study, AflN was identified in *A. flavus* by alignment of protein sequence with AflN in other fungi. Phylogenetic tree analysis of AflN indicated that *A.flavus* AflN showed high homology with AflN in *A.oryzae* and *A.parasiticus* (Figure 1A,B), suggesting a potential function in the conversion of VA to ST, a step of AF biosynthesis. To study the function of AflN in *A.flavus*, Δ*aflN*, *aflN-com* and *OE::aflN* strains were successfully constructed with homology recombinant method. These mutant strains were confirmed with PCR, and *aflN* expression were also quantified using qRT-PCR in various strains, which indicated that Δ*aflN*, *aflN-com* and OE::*aflN* strains were successfully constructed (Figure 1D,E).

For *A. flavus*, AF biosynthesis is one of the main characteristics, which were under intense investigation. During the biosynthesis, the conversion of VA to ST is one of complicated and undefined steps, in which AflY, AflX, and AflM are all involved [16,17,18]. In *A. nidulans*, *stcS*, the homologues of *aflN*, was suggested to be involved in the conversion of VA to ST [9]. In this study, AflN was found to play a role in AF biosynthesis (Figure 2A), consistent with the function of the homology StcS in *A. nidulans*. As the homology of StcS, the absence of AflN possibly causes the defect of the formation of ST, leading to the undetected level of AF, which suggests a direct role of AflN in AF biosynthesis in *A.flavus*. AflN distribution is important for its function. As shown in Figure 2B, our result indicated that AflN is located at the cytoplasm. The location of AflN is consistent and stationary at 8 h and 24 h, suggesting the function of AflN will be concentrated at cytoplasm. As a member of cytoplasmic P450 superfamily, AflN possibly plays its role at the cytoplasm [19,20]. Consistently, AflN was involved in aflatoxin biosynthesis (Figure 2), which is mainly taking place in cytoplasm [7,21]. Moreover, aflatoxisomes (aflatoxin-synthesizing vesicles) were proved to be units capable of synthesizing aflatoxins including AFB1 [22,23]. Ver-1 has been found in cytoplasm, vesicles and vacuoles in *A.parasiticus* [22]. Thus, as a middle enzyme of AFB1 biosynthesis, it is possible that AflN is also located at aflatoxisomes for aflatoxins synthesis in *A. flavus*. Interestingly, AflN tends to aggregate at undefined places in hypal cells (Figure 2B). To assume that these undefined places partly overlap with aflatoxisomes is reasonable, although it needs further confirmation. Therefore, our results suggested that AflN located in cytoplasm plays its role in the AF biosynthesis in *A. flavus,* beneficial to the further function study of AflN. Moreover, our results provide the genetic evidence for the role of *aflN* in the AF biosynthesis in *A. flavus*.

As known, conidia are the important form for the spread of *A. flavus* [24]. Our study also found that AflN is involved in the conidiation. The poorly developed conidiophores and decreased *brlA* expression were also observed in Δ*aflN*. Considering that conidia development is mainly regulated by *brlA* [11], this impairment of conidiation is possibly due to the decreased expression of *brlA* in Δ*aflN*. Moreover, BrlA is necessary and sufficient for conidiophore development [11]. Therefore, decreased *brlA* expression plays a crucial role in the abnormal development of conidia in Δ*aflN*. However, it is impossible to ignore the accumulation of versicolorin A, the substrate of AflN [8], and the absence of AFB1 (Figure 2A) in Δ*aflN*. Although no evidence suggests that versicolorin A inhibit conidiophore, the possibility cannot be excluded. At the same time, reduced AFB1 is always associated with poor conidiophores and impaired conidiogenesis [6,25]. Although the detailed mechanism may be different, AFB1 seems to be a beneficial signal for conidiogenesis. The roles of AflN in hyphal growth and sclerotia were also investigated in this study. Our results demonstrated that AflN has no effect on the hyphal growth and sclerotial development in *A.flavus* (Figure 4). All together, these results indicated that AflN plays an important role in conidiation but not in hyphal growth and sclerotia development.

During the vegetative growth, *A. flavus* undergoes and responds to various environmental stresses, which may be closely related to AF biosynthesis [26]. In this study, the inhibiting effect of oxidative stress on *A. flavus* was decreased in Δ*aflN* (Figure 5A,B). At the same time, the increased ROS has been observed in Δ*aflN* (Figure 5C,D)*,* which poses *A. flavus* the state of anti-ROS for the balance of oxygen-reducing system. The increased ROS may be partly due to the decreased ROS scavenging enzymes, catA and catalase-like in Δ*aflN* (Figure 5E), but this does not exclude other reasons. It is worth noting that aflatoxins biosynthesis also has close relationship with ROS. It was proposed that aflatoxin biosynthesis is part of the cellular response to oxidative stress [27,28]. High level of ROS is able to trigger aflatoxin biosynthesis associated with increased expression of aflatoxin cluster genes [28]. The upregulation of gene expression is regulated by transcription factors including AtfB, SrrA, AP-1 and MsnA, which are triggered by the increased ROS [28]. *fas-1*, *omtA* and *ver-1* in AF gene cluster were proved to respond to the regulation factors [28]. Interesting, upregulation of *aflP* (*omtA*) and *aflM* (*ver-1*) have been observed in Δ*aflN* (unpulished data), although related transcriptional factors have not been examined. How the absence of AflN affects the expression of ROS scavenging enzymes is unknown and this demands further study. All of these results indicated that *aflN* is involved in anti-oxygen stress in *A. flavus*.

The AFB1 contamination of food is usually concerned with prevention and control of *A. flavus* in agriculture. In this study, AFB1 were undetected in peanuts and maize infected with Δ*aflN* (Figure 6), significantly decreasing the virulence and pathogenicity of *A.flavus*. At the same time, conidia number was decreased in Δ*aflN* infection, which will restrict the dispersion of *A.flavus* [29]*,* compared with WT. The impaired pathogenicity in Δ*aflN* is possibly due to the role of AflN in AFB1 biosynthesis and conidiation. These results also suggested that study of *aflN* function has the potential value in the prevention and control of *A. flavus* and aflatoxin contamination.

## 4. Conclusions

In conclusion, *aflN* is involved in the AF biosynthesis, oxidative stress response, conidiation and pathogenicity, but not in growth and sclerotia formation. These findings contribute to the better understanding of *aflN* functions in *A. flavus*, which potentially helps to provide a reference for scientific prevention and control of *A. flavus*.

## 5. Materials and Methods

### 5.1. Mycelia Growth, Conidiation and Sclerotia Production

*A. flavus* strains were listed in Table 1. For mycelia growth assays, various strains were cultured on solid YES (yeast extract-sucrose) and PDA (potato dextrose agar) at 37 °C for indicated time. Then colony diameters were observed and measured [30]. For conidia assay, YGT (yeast extract, glucose and trace elements) and PDA were used [6]. For sclerotial production analysis, strains were grown on solid CM (complete medium) at 37 °C for 7 days in the dark [6].

### 5.2. Construction of Gene Mutants

The *aflN* deletion (Δ*aflN*), complementary (*aflN-com*) and overexpression (*OE::aflN*) strains were constructed with the methods previously described [6,25,31]. Briefly, the upstream (AP, A homology arm part) and downstream (BP, B homology arm part) sequences of *aflN* gene, as well as the *pyrG* gene from *A. fumigatus,* were fused into an interruption fragment (AP-*pyrG*-BP) using fusion PCR strategy. The AP fragment was amplified using primers *aflN-a1* and *aflN-a3*, while the BP fragment was amplified using primers *aflN-b6* and *aflN-b8*. *pyrG* gene was amplified using primers *pyrG-F* and *pyrG-R*. *aflN-b2* and *aflN-b7* are nesting primers for further amplifing AP-*pyrG*-BP fragment, which is from the overlap extension PCR of AP, BP, and *pyrG*. The fused fragment (AP*-pyrG*-BP) was then transformed into *A. flavus* CA14 PTs protoplasts to construct *aflN* knockout strain (Δ*aflN*). The Δ*aflN* strain was primarily confirmed with PCR method. AP, BP and ORF (open reading frame for aflN) fragments were amplified using primers *aflN-a1*/*P801-R*, *P1020-F*/*aflN-b8*, and *aflN-F4*/*aflN-F5*, respectively, to validate if the insertion takes place at the designed site. For the construction of complementary strain (*aflN-com*), two steps of homology recombination were used. First, the *pyrG* gene in Δ*aflN* was replaced with *aflN* gene fragment, which is confirmed by PCR method. Second, *pyrG* gene was inserted into the site between *aflN* ORF and 3′UTR of the intermediate strain for the selection of *aflN*-com strain. For the construction of overexpression strain (*OE::aflN*), *aflN* gene with *gpdA* promoter from *A. nidulans* was amplified, and the fused fragment (AP-*pyrG*-*gpdA*-BP) was transformed into A. flavus CA14 PTs protoplasts to construct *OE::aflN*. All mutant strains were confirmed by PCR, sequencing, and qRT-PCR. Primers used for *aflN* mutant strains construction and verification are listed in Table 2. For the localization of AflN, *aflN-gfp* fusion cassette was constructed as previously described [32].

### 5.3. The Levels of Intracellular ROS

The intracellular ROS level was measured by using a ROS assay kit (Beyotime Institute of Biotechnology, Shanghai, China) with the protocol manual. In brief, the harvested mycelia were incubated with PBS (phosphate buffered saline) containing 10 μM DCFH-DA (6-carboxy-2,7-dichlorodihydrofluorescein diacetate) for 30 min. The fluorescence signal of ROS level was acquired by confocal microscope.

### 5.4. Stress Analysis

Various strains in this study were pointed onto solid PDA supplemented with oxidative stress-inducing reagents (1 mM or 2.5 mM H_2_O_2_). Then the plates were incubated at 37 °C in the dark for 4 days before the calculation of relative inhibition.

### 5.5. AF Analysis

AF was extracted from liquid YES medium using chloroform [33]. TLC was used to analyze the level of AF biosynthesis, as previously reported [30]. In brief, 5 μL of AF suspension was loaded into a silica gel plate and separated by chromatographic solution including acetone: chloroform (1:9, *v/v*). Silica gel plates were examined with Gel Doc XR+(Bio-Rad) and captured at 312 nm wavelength. The AFB1 signal was analyzed and quantified with Image J.

### 5.6. The Infection of Peanuts and Maize Seeds

To assess the pathogenicity of *A. flavus*, peanut and maize seeds, which were kept in our lab and used for the infection, were washed with sodium hypochlorite, ethanol and sterile water. The washed seeds were then infected by immersing in a 10^7^ spores suspension for 30 min. Then, the seeds were placed in culture dishes lined with moist sterile filter paper for 5 days. The infected seeds were pictured and collected for spores count and AFB1 quantification.

### 5.7. qRT-PCR

Total RNA was prepared from the mycelia of *A. flavus* with the use of RNA extraction kit (TIANMO BIOTECH, Beijing, China). SYBR Green Supermix (Takara) was used for the qRT-PCR reaction with the PikoReal 96 Real-time PCR system. The 2^−ΔΔCT^ method was used to quantify the expression levels of the indicated genes [34]. PCR primers for qRT-PCR were shown in Table 3.

### 5.8. Statistical Analysis

Data are presented as means ± SD. All statistics were performed using Student’s *t* test with SPSS 11.5 software. A *p* < 0.05 was considered statistically significant.

## Figures and Tables

**Table 1:** *A. flavus* strains used in this study.

Strain	Genotype Description	Reference
*CA14 PTS*	∆*ku70*, Δ*niaD*, ∆*pyrG*	[31]
*wild-type*	Δ*ku70*, Δ*niaD*, Δ*pyrG*:: *pyrG*	This study
Δ*aflN* *aflN-com*	Δ*ku70*, Δ*niaD*, ∆*pyrG*, Δ*aflN*:: *pyrG* Δ*ku70*, Δ*niaD*, Δ*pyrG*::*aflN*, *pyrG*	This study This study
*OE::aflN*	Δ*ku70,* Δ*niaD*, Δ*pyrG*::Gpda(p)-*aflN*, *pyrG*	This study
*aflN-gfp*	Δ*ku70,* Δ*niaD*, Δ*pyrG*:: *aflN*-*gfp*, *pyrG*	This study

**Table 2:** Primers for strains construction.

Primer Name	Sequence(5′→3′)	Application
*aflN-a1*	AGGTATTCAGATATTTCGGTCTC	For Δ*aflN* construction and verification
*aflN-a3*	GGGTGAAGAGCATTGTTTGAGGCGGTCATGTCCCTAGTTCGT	
*aflN-b6*	GCATCAGTGCCTCCTCTCAGACATTTGTAAGAATGTCGTGCCT	
*aflN-b8*	GTCGCGGGAGGAAATGA	
*aflN-a2*	ATCCTGACCAGCTCTAA	
*aflN-b7*	CCTTTCCAAACCCTAC	
*aflN-F4*	TTCCTGACGGCGTTCTA	
*aflN-R5*	CACGATGCCCATTGACTT	
*com-aflN-F*	GCAGCCACCCAAATACAAAAGT	For *aflN-com* construction and verification
*com-aflN-R*	GGGTGAAGAGCATTGTTTGAGGCCATGACCCTCACTAAAACTACCCT	
*P1020-F*	ATCGGCAATACCGTCCAGAAGC	
*P801-R*	CAGGAGTTCTCGGGTTGTCG	
*pyrG-F*	GCCTCAAACAATGCTCTTCACCC	
*pyrG-R*	GTCTGAGAGGAGGCACTGATGC	
*qPCR-aflN-F*	TCCTCTCGAGTCGCTCACCAC	
*qPCR-aflN-R*	ACCATAGTACCAACGGCCTAA	
*gpdA-F*	GCATCAGTGCCTCCTCTCAGACGAGGACTGCAATCGCCATGAGGTTT	For *OE::aflN* construction and verification
*gpdA-R*	CAAGCTGCGATGAAGTGGGAAAG	
*aflN-OE-AF*	AGTGGTTGAACAGATCAAGGC	
*aflN-OE-AR*	GAAGAGCATTGTTTGAGGCCTATACATCGTCAGCTTCAGGA	
*aflN-OE-BF*	CAAAGAGCAAACCTTCCTATGCCAGAGTTCAAGCT	
*aflN-OE-BR*	TGAGAACAGGAGATAGACAGC	
*aflN-OE-MF*	CGCTTGAGCAGACATCACAATGTACCTTTCGCTCCTCAT	
*aflN-OE-MR*	GAACTCTGGCATAGGAAGGTTTGCTCTTTGCAGC	
*aflN-OE-P2*	GAGGCCTATCGCCATATGCG	
*aflN-OE-P7*	CACTCATCGTATGCTGGCG	

**Table 3:** Primers for qRT-PCR.

Primers Name	Sequence	Application
*brlA-F*	GCCTCCAGCGTCAACCTTC	*brlA*
*brlA-R*	TCTCTTCAAATGCTCTTGCCTC	
*nsdD-F*	GGACTTGCGGGTCGTGCTA	*nsdD*
*nsdD-R*	AGAACGCTGGGTCTGGTGC	
*Catalase-F*	TCGAACAATTCCGTGGTATG	*Catalase like*
*Catalase-R*	AGCTGGTCGCTCCCGATGGA	
*CatA-F*	CGCCATCATTATCGGCGACGGA	*CatA*
*CatA-R*	TGAGGCTTTCGACGTGCGGAC	
*β-tublin-F*	TTGAGCCCTACAACGCCACT	*β-tublin*
*β-tublin-R*	TGGTTCAGGTCACCGTAAGAGG	
*Actin-F*	ACGGTGTCGTCACAAACTGG	*Actin*
*Actin-R*	CGGTTGGACTTAGGGTTGATAG	
